# Correction of lesser toe deformities: minimally invasive versus open surgery—a prospective randomised study

**DOI:** 10.1007/s00402-025-06114-1

**Published:** 2025-11-24

**Authors:** Benjamin Weigang, Angelina Garkisch, Angela Simon, Thomas Mittlmeier

**Affiliations:** 1 Diakonie Klinikum Dietrich-Bonhoeffer Malchin, Malchin, Germany; 2 Universitätsmedizin Rostock, Rostock, Germany

**Keywords:** Lesser toe deformities, Minimally invasive surgery, Open surgery, Safety, Outcome, Hammertoe, Claw toe

## Abstract

**Objective:**

To compare soft and hard outcome measures after minimally invasive (MIS) and open (OS) surgical treatment of lesser toe deformities. It was hypothesised that MIS would be associated with fewer complications and comparable subjective and objective results.

**Methods:**

A prospective randomised controlled study was designed. One hundred patients were included consecutively and allotted via block randomisation to two groups. The presence of co-pathologies at the forefoot and midfoot, which were treated simultaneously, was not an exclusion criterion. However, these cases were analysed to ensure an equal distribution across the study groups. Patients were evaluated clinically, functionally, and radiologically prior to surgery and at a 1.5-year follow-up. Additionally, they were asked about their personal satisfaction via patient-reported outcome measures.

**Results:**

The distribution of co-pathologies at the foot was not significantly different between the two study groups. The same is true of co-morbidities. Significantly more wound complications, including infections, were found in the open surgery group (*p* = 0.029). K-wire issues were distributed equally between the groups, but differed in their clinical manifestation (*p* = 0.03). Only seven out of the 95 patients finally examined were dissatisfied with their long-term results, with an equal distribution between both groups (four MIS vs. three OS, *p* = 0.914). Clinical and radiological corrections of the lesser toes were comparable in both groups, but the open surgery (OS) group showed significantly more non-unions (*p* = 0.0013). Functional evaluation via the FFI-D (Foot Function Index Germany), a reliable, validated, internationally used, standardised questionnaire to assess the correlation between foot deformity and function, demonstrated relevant postoperative improvement in all patients, with no difference between the two technical approaches (*p* = 0.460).

**Conclusion:**

Lesser toe surgery is a low-risk treatment with good overall results. MIS offers equivalent clinical outcomes to OS with a lower risk of complications in terms of soft tissue and bone healing.

**Level of evidence:**

Level 1 Prospective randomised controlled study. TRN DKRS00034137 25/04/2024.

## Introduction

 Lesser toe deformities are common and include mallet, hammer, claw and curly toe deformities [1]. Over time, different surgical techniques have been developed to accomplish the main goals of lesser toe surgery: creating physiological and functional positioning of the treated ray, while maintaining the stability, flexibility, and mobility of the lesser toe, to achieve a pain-free, powerful gait.

Traditionally, open surgery has been used to treat lesser toe deformities for decades. In principle, three basic options have been described, including soft tissue procedures (extensor tendon lengthening, dorsal capsulotomy and metatarsophalangeal joint release, and plantar plate repair), tendon transfers [2]. The more recently established range of minimally invasive techniques includes soft tissue procedures such as flexor and extensor tenotomies and plantar capsular release of the metatarsophalangeal (MTP) or proximal interphalangeal (PIP) joint, as well as bony procedures such as proximal and middle phalanx osteotomies, condylectomy and DIP and PIP joint arthrodesis, and combinations thereof [2, 3]. The decision-making process for selecting the percutaneous surgical approach has been developed according to a decision tree based on the degree of rigidity of the deformity [2].

So far, little data has been presented on the comparison between established open surgery and the growing minimally invasive treatment of lesser toe deformities [3–6].

This randomised controlled study was therefore performed with the primary aim of evaluating the safety of these two surgical techniques (OS vs. MIS). The secondary aims were to determine patient satisfaction in both study groups and the corresponding clinical, functional and radiological outcomes. It was hypothesised that MIS treatment would be associated with fewer complications than OS treatment, while producing comparable subjective and objective results.

## Methods

The study was approved by the local ethics committee (registration no. A 2020-0247) and adhered to the principles of the Declaration of Helsinki and the Guidelines for Good Clinical Practice. The study protocol was registered with the German Clinical Trials Register (DRKS00034137), which is accessible without restrictions.

A total of 100 patients of all sexes over 18 years of age were recruited for the prospective, randomised, monocentric clinical trial. These patients had visited the outpatient unit with solitary or combined lesser toe deformities. Potential participants received written information about the trial design, aims and specific objectives when the options for therapeutic interventions were explained, prior to scheduling surgery. Informed written consent to participate in the study was a prerequisite. Patients unable to comply with the requirements of the study were excluded. Additional planned simultaneous forefoot and midfoot procedures, such as hallux valgus or hallux rigidus surgery, or fusion for TMT joint instability, did not lead to exclusion, but were analysed for equal distribution between the study groups (Table 2). Patients were included in the study consecutively under the supervision of the principal investigator. Block randomisation paired with sequentially numbered, opaque, sealed envelopes (SNOSE) was used to allocate participants to the two study groups. Fifty patients were assigned to MIS and the remaining 50 patients formed the OS group (Fig. 1 flowchart). The two groups were comparable in terms of demographics and relevant comorbidities, such as diabetes mellitus or neuropathy (Table 1). Surgeries were performed from December 2020 until April 2023. Based on our observation that results after lesser toe surgery reach a steady state after 12–18 months, the final follow-up examinations were performed after 1.5 years. Basic data and clinical follow-up data were collected in an unblinded manner, while the radiographic analysis was performed by blinded examiners. All stages of the trial took place in the Department of Foot and Ankle Surgery at the institution of the corresponding author. Patient data was pseudonymised and stored on a computer dedicated solely to the study. Access to the data was limited to the principal investigator. CONSORT reporting guidelines were used to meet the scientific requirements of a prospective randomised clinical trial [7] (Figs. 2, 3, 4, 5, 6 and 7).

**Fig. 1:**
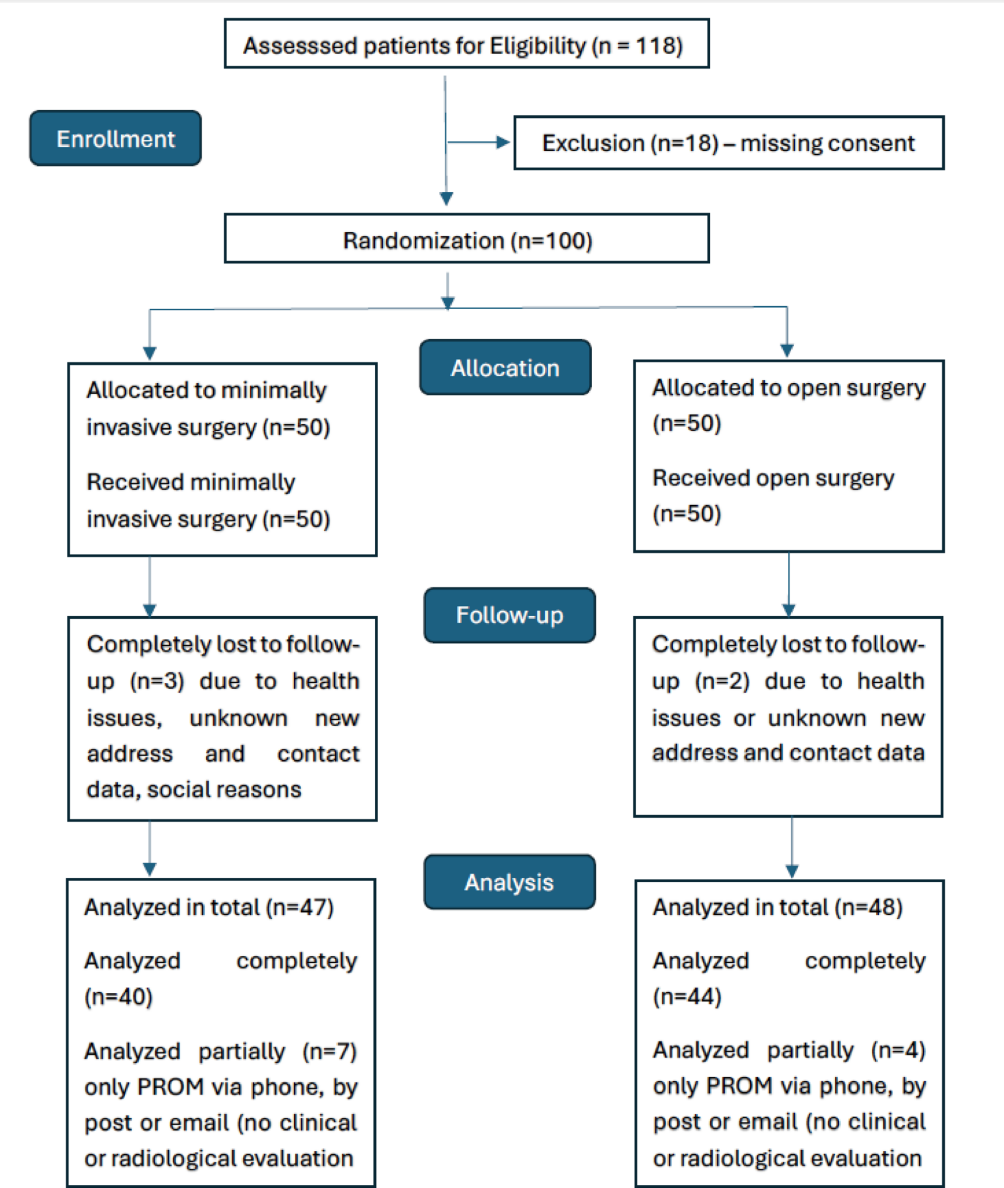
Study design

**Fig. 2:**
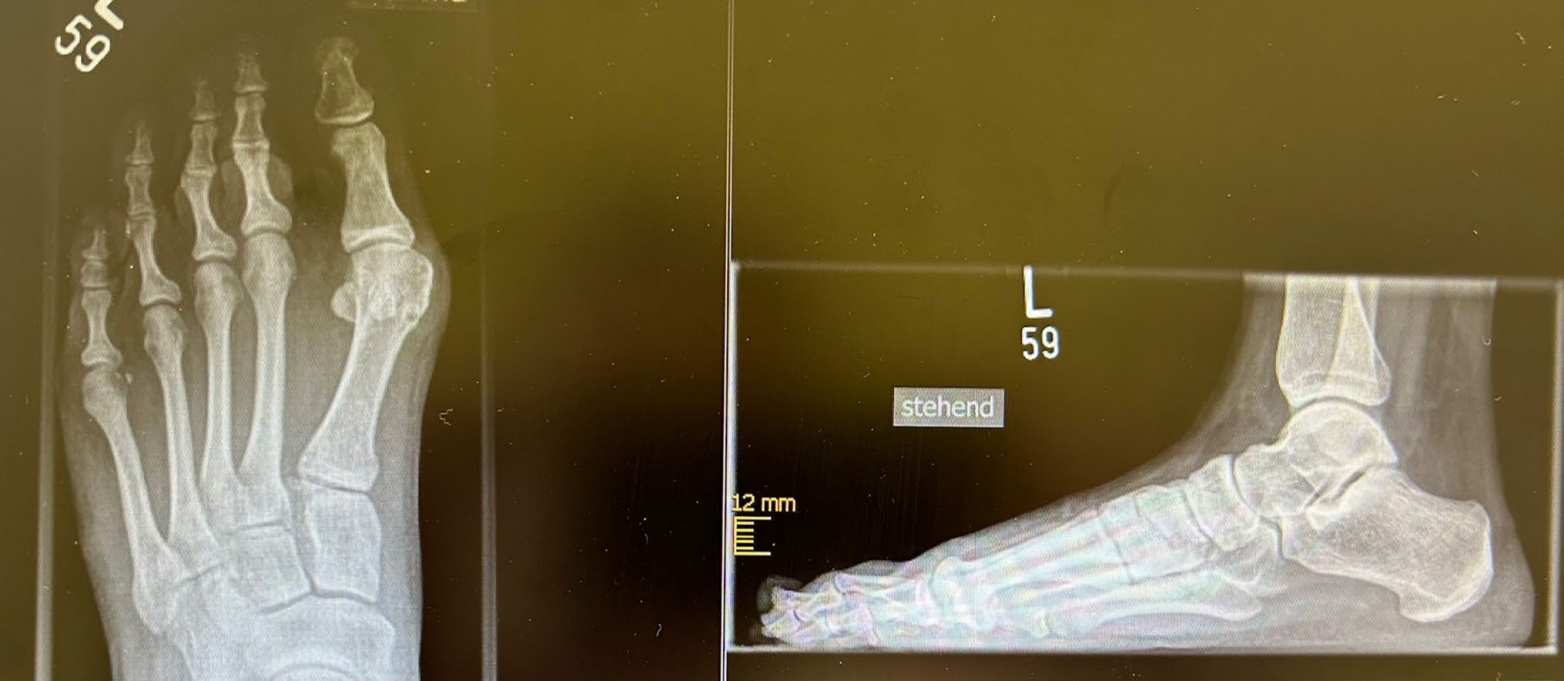
X-ray: preoperative Hammertoe left Digitus pedis II

**Fig. 3:**
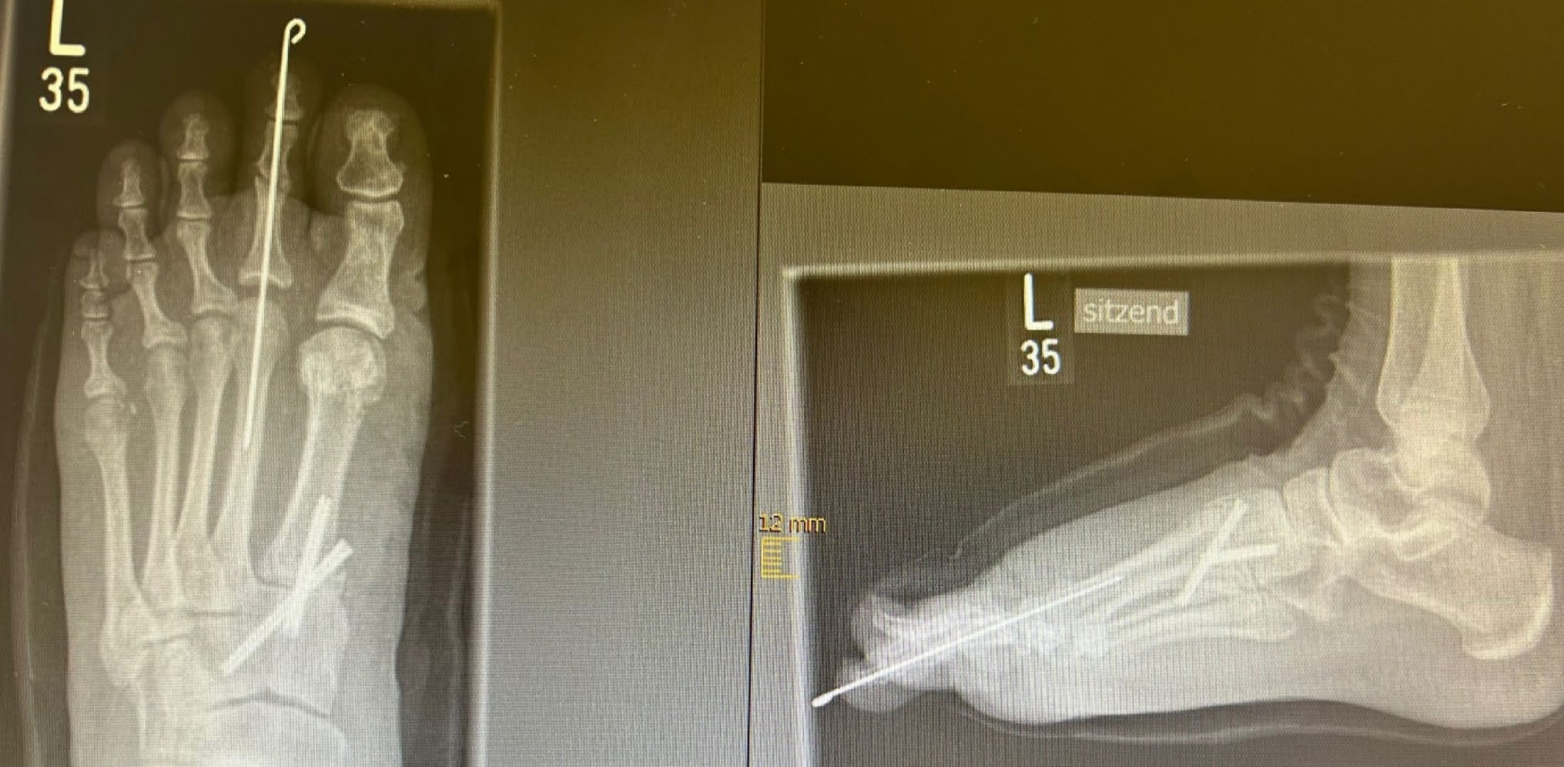
X-ray: postoperative results after open surgical correction of the 2nd ray

**Fig. 4:**
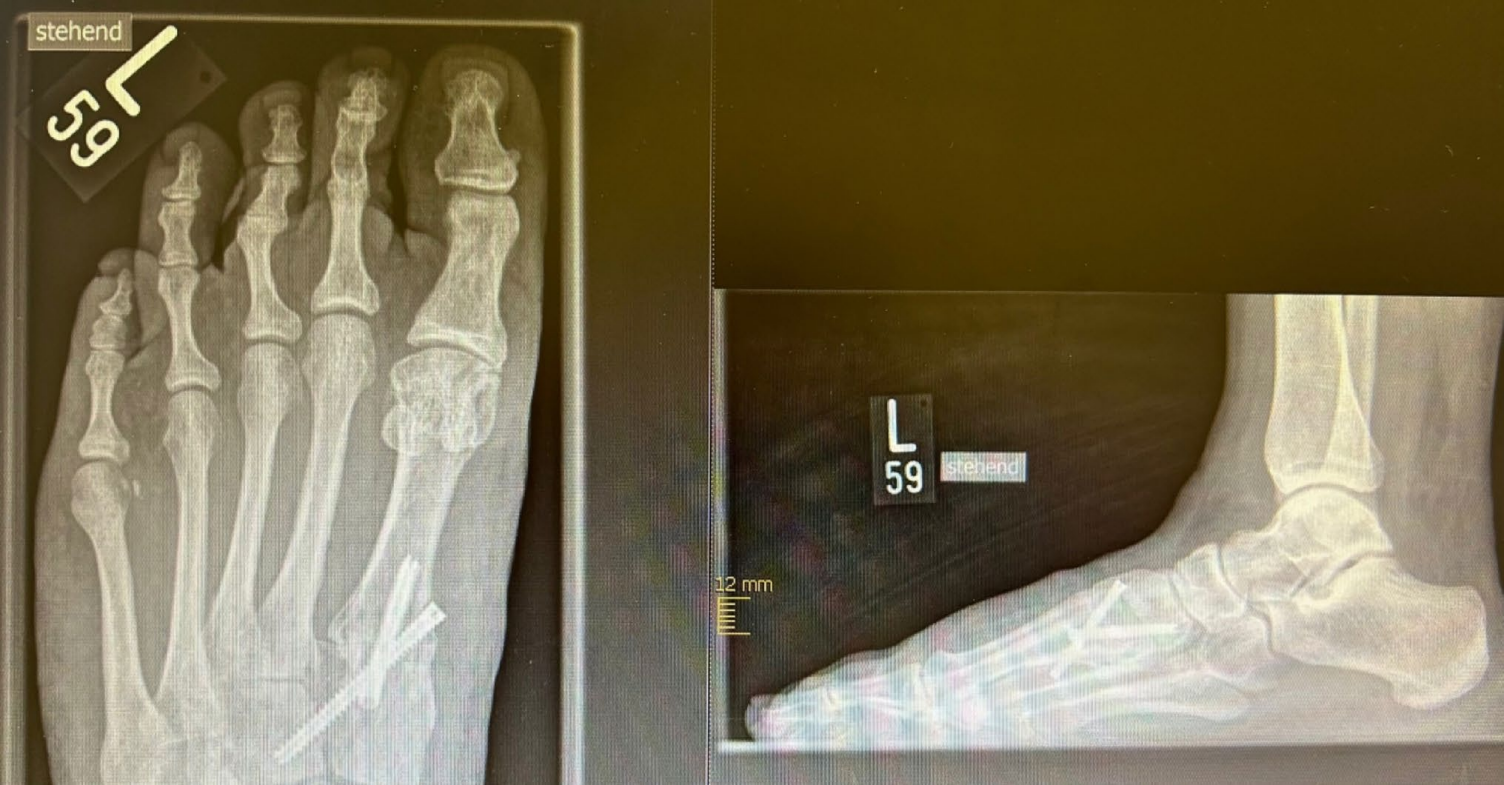
X-ray: long-term results after OS

**Fig. 5:**
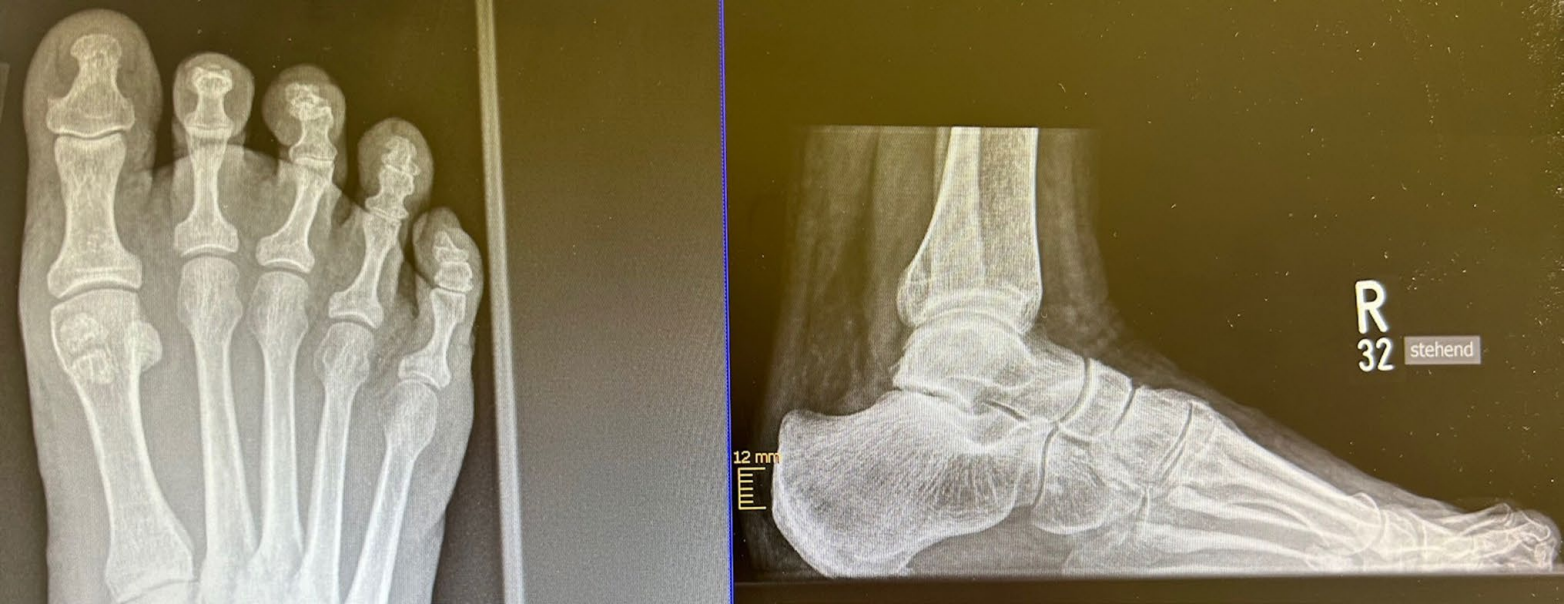
X-ray: preoperative Hammertoes right Digiti pedis II and III

**Fig. 6:**
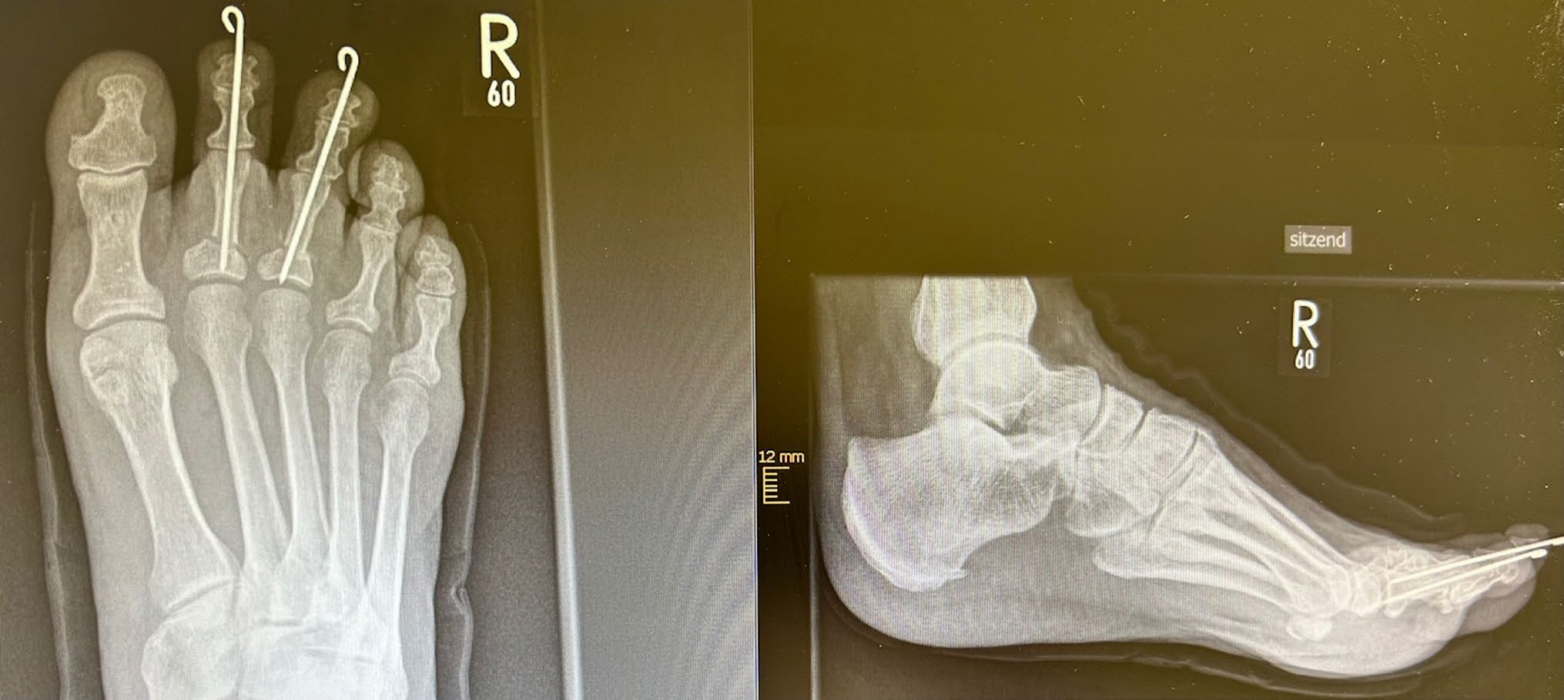
X-ray: postoperative results after minimally invasive correction of the 2nd and 3rd ray

**Fig. 7:**
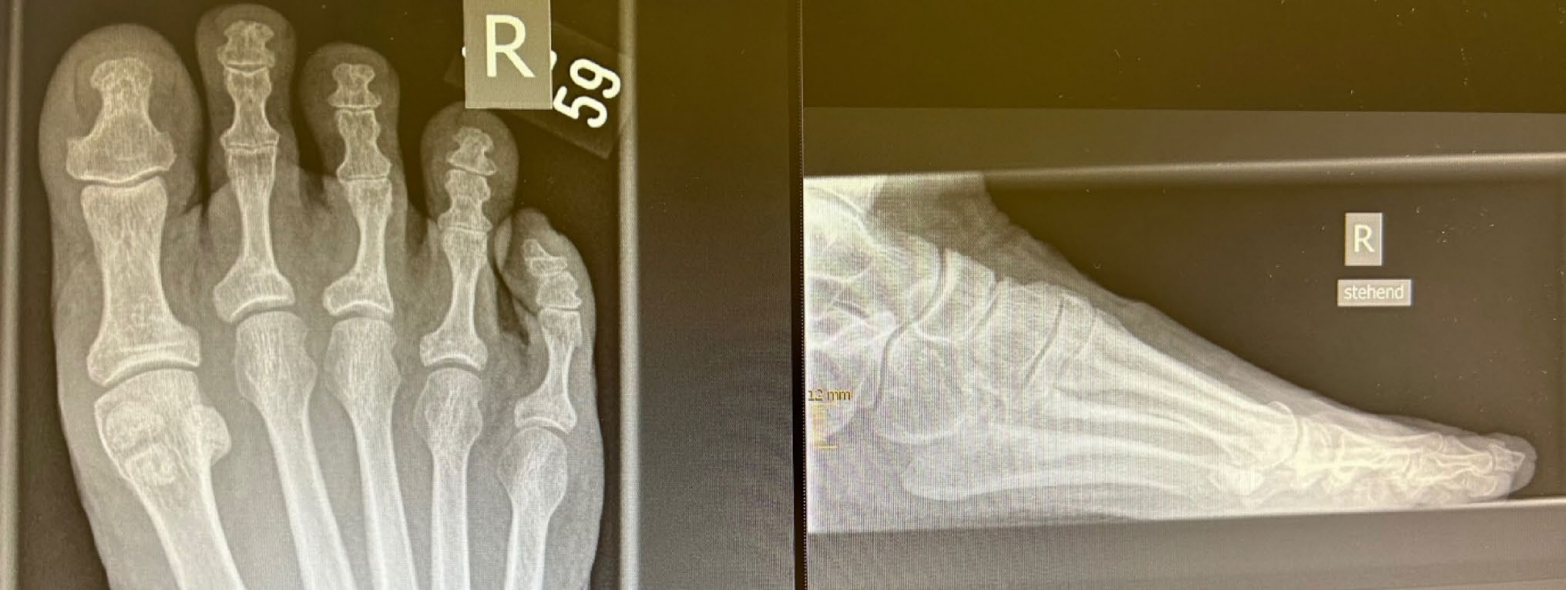
X-ray: long-term results after MIS

**Table 1:** Demographic data of the two groups

	Minimally invasive surgery (MIS) Mean ± SD (CI)	Open surgery (OS) Mean ± SD (CI)	Test statistics	*p* value
Number of Patients	47	48		
Age (y)	60.6 ± 9.2 (58.41–64.29)	62.9 ± 11.5 (60.4-67.24)	U = − 0.931	0.352
Gender	n %	n %	χ² = 0.407	0.524
Female	36 76.6	34 70.8		
Male	11 23.4	14 29.2		
Weight (kg)	79.0 ± 18.6 (74.32–86.43)	77.0 ± 10.2 (73.78–81.31)	U = − 0.443	0.658
Height (cm)	168.7 ± 10.5 (165.68-172.22)	168.8 ± 10.5 (165.8-172.11)	U = − 0.194	0.846
Follow-up (days)	474.9 ± 179.3 (444.35-564.14)	535.3 ± 153.8 (481.16-576.76)	U = − 1.098	0.272
Type of Lesser toe deformity (n)	47	48	U = − 2.192	0.169
Hammer toe Patients (n)	42	34	χ² = 6.29	0.012
Claw toe Patients (n)	4	13	χ² = 5.1	0.024
Mallet toe Patients (n)	1	1	χ² = 0.00024	0.99
Metatarsal Procedures (n) Diabetes mellitus (n) Neuropathy	0 7 2	1 2 1	χ² = 0.98 χ² = 2.91 χ² = 0.35	0.32 0.088 0.55

Significance at *p* < 0.05

Mean ± Standard deviation

*CI* 95% Confidence interval, *U* Mann–Whitney U, *χ²* Chi-square

**Table 2:** Relationship between surgical approach (minimally invasive vs. open) and concomitant pathologies in the mid- or forefoot

Co-pathologies	MIS (*n* = 47)	OS (*n* = 48)	Total (*n* = 95)	Test statistics	*p* value
No comorbidity	13	7	20		
Hallux valgus left	4	4	8		
Hallux valgus right	7	8	15		
Hallux rigidus left	1	2	3		
Hallux rigidus right	4	0	4		
TMT-I instability left	7	9	16	χ² = 11.16	0.43
TMT-I instability right	9	10	19		
Tailor’s bunion right	1	2	3		
Previous surgery 1st ray right	0	1	1		
Pes planovalgus right	0	2	2		
Lisfranc osteoarthritis I-III left	0	1	1		
Total	47	48	95		

Significance at *p* < 0.05

*χ²* Chi square, *MIS* minimally invasive surgery, *OS* open surgery, *TMT* tarsometatarsal joint

### Surgical techniques

Minimally invasive correction was achieved by percutaneous release of the plantar capsule of the proximal interphalangeal joint and disintegration of the short flexor, in combination with a percutaneous plantarising closing wedge osteotomy of the proximal phalanx, with optional release of the metatarsophalangeal joint, and a dorsal closing wedge osteotomy of the middle phalanx if necessary. Retention was secured by a 1.4 mm retrograde longitudinal K-wire transfixion into the base of the proximal phalanx. In cases of metatarsalgia, an additional distal minimally invasive metatarsal osteotomy (DMMO) was optional. Open surgical correction was mostly performed using a Z-split of the long extensor tendon for planned lengthening and a balanced release of the MTP joint. This was followed by a V-shaped arthrodesis of the proximal interphalangeal joint with retrograde longitudinal K-wire transfixion into the metatarsal bone. If necessary, a subcapital elevating osteotomy of the metatarsal was performed with screw fixation. Both groups received K-wire transfixation for all types of deformity.

The postoperative regimen was identical for both groups. Redressement of the toes via taping was not applied as the correction was held with K-wires. Patients were mobilised using a specific boot (Dual Relief, Darco (Europe) GmbH, Germany), with a recommendation for four weeks of partial weight-bearing using crutches. This was practised under the supervision of a physiotherapist during the in-hospital stay. Full weight-bearing was permitted following the removal of the K-wires, four weeks after surgery. Manual lymphatic drainage and physical therapy were optional, depending on individual needs.

### Patient follow-up

Preoperatively, baseline information was gathered, such as demographics, health conditions, history of foot treatments, mechanical footwear issues, type of deformity, and risk factors, using case report forms. Functional status was determined using the Foot Function Index Germany (FFI-D), a 23-item questionnaire with proven reliability and validity [8]. Final scoring was achieved by summing each patient’s responses, which ranged from 1 to 9 per item. Standard radiography was performed prior to the intervention, postoperatively, and during the follow-up examination after 18 months. Osseous healing was assessed using two radiographic views to confirm the presence of bridging callus or bone, as previously described [9]. After the operation, patients were evaluated for compliance and clinical course. Any complications were documented, e.g. ischaemia, prolonged wound healing, surgical site infection, K-wire issues, or deep vein thrombosis. Patients were asked about their personal satisfaction with the surgical results, their willingness to undergo the procedure again and their willingness to recommend the treatment to other patients or family members via a patient-related outcome measurement questionnaire. Furthermore, a clinical evaluation of lesser toe correction in all three dimensions was performed. Clinical assessment of toe alignment was subdivided into three classes (complete, satisfactory or inadequate). Eighteen months after surgery, clinical and radiological control focused on long-term complications such as scarring, strictures, arthrofibrosis, neuropathic pain or dysesthesia, recurrent deformity, non-union or instability. As blinded examination was impossible due to the manifest scars indicating the type of surgery performed, the clinical assessment was non-blinded. Radiographical alignment was classified into three categories (good, tolerable or inadequate).

Function and satisfaction questionnaires were completed during the final follow-up examination.

### Statistical analysis

Statistical analysis was performed using IBM SPSS Statistics for Windows, Version 29.0.2.0 (IBM Corp., Armonk, NY, released 2023). Data were primarily analysed using descriptive statistics to display the mean, median and standard deviation. Variables were tested for normal distribution using the Kolmogorov–Smirnov test. If normal distribution was absent, nonparametric tests were applied. Significance tests on independent ordinal variables were performed using the Mann–Whitney U-test. Associations between categorical variables were investigated using Pearson’s chi-squared test (cross tables). Dependent ordinal variables were evaluated using the Wilcoxon test. The significance level was set at *p* < 0.05.

## Results

The preoperative demographic characteristics are given in Table 1. There were no demographic differences between the two study groups or in the follow-up period (*p* > 0.05). The majority of patients were female. Relevant comorbidities, such as diabetes mellitus or peripheral neuropathy, were found asymmetrically within the study groups, but no statistical difference was observed (Table 1). Lesser toe deformities were distributed similarly within the two study groups, except for claw toes and transverse instabilities, which were found more frequently in the OS group. There were 11 cases of transverse instability in the OS group and 3 cases in the MIS group, resp. The number of lesser toes involved was equally distributed between the two groups (*p* = 0.92). There was one case of metatarsal elevating osteotomy in the OS group. Co-pathologies addressed during the index surgery were not statistically unevenly distributed between the study groups (Tables 2).

Of the original 100 patients (50 in each group), 95 were re-evaluated at the 18-month follow-up. Five patients could not be included in the follow-up due to a change of address, serious health issues, and one case of death unrelated to surgery. Forty-seven patients received MIS and 48 patients had OS. Eleven of these individuals could only be re-evaluated via telephone or email contact (PROM questionnaires). Consequently, a clinical and radiological assessment was not possible for these individuals. Complete data sets were available for 84 individuals, corresponding to an 84% retrieval rate. At the final follow-up, overall patient satisfaction, as shown in Table 3, was high (88.4% satisfied and 4.2% undecided), with no significant difference between the two surgical techniques (*p* = 0.914). The functional outcome (FFI-D), as shown in Table 4, was also comparable in both cohorts (*p* > 0.05). The number of painful days and total FFI-D scores improved significantly for both surgical techniques when preoperative and postoperative values were compared (*p* < 0.001). Table 5 shows the clinical evaluation at the final follow-up regarding the three-dimensional correction of lesser toe deformities and the presence of persistent shoe conflict. Both minimally invasive and open surgeries permitted a high overall correction rate, with only four toes in four out of 84 patients exhibiting recurrent deformities. Misalignments were equally distributed between the MIS (two toes in two patients) and OS (two toes in two patients) groups, displaying no significant clinical difference (*p* = 0.833). The radiological outcome of both groups is shown in Table 6. Eighteen months postoperatively, the MIS group exhibited a 100% osseous consolidation rate, whereas the OS group demonstrated complete bony healing in only 36 out of 44 patients (58 out of 66 toes). Two patients (two toes) showed partial osseous consolidation (*p* = 0.029). Six patients in the OS cohort presented with a manifest non-union (six toes), with no such cases in the MIS group (*p* = 0.013). Anatomic axial correction was achieved in both groups (*p* = 0.145), with two cases of insufficient correction in each cohort. The complications in both cohorts are given in Table 7. There were significant differences in wire complications between the two groups (*p* = 0.03), with four wire migrations in the MIS patients and three wire deformations in the OS patients. The OS group had significantly higher rates of impaired wound healing (*p* = 0.029) and wound infection (*p* = 0.023). There were no floating toes. No toes were lost due to ischaemic complications.

**Table 3:** Patient satisfaction 18 months postoperatively

	Minimally invasive surgery (MIS)	Open surgery (OS)	Test statistics	*p* value
Satisfaction (yes/indecisive/no)	41/2/4	43/2/3	χ² = 0.180	0.914
Will to repeat the procedure (yes/indecisive/no)	42/1/4	40/2/6	χ² = 0.772	0.680
Recommendation to patients (yes/indecisive/no)	42/1/4	41/2/5	χ² = 0.446	0.800
Recommendation to family (yes/indecisive/no)	42/1/4	41/2/5	χ² = 0.446	0.800

Significance at *p* < 0.05

*χ²* Chi square

**Table 4:** FFI-D preoperatively and follow-up

	Minimally invasive surgery (MIS) Mean ± SD (CI)	Open surgery (OS) Mean ± SD (CI)	Test statistics	*p* value
Number of painful days the past week - preoperatively	4.5 ± 2.5 (3.7–5.3)	4.9 ± 2.5 (4.1–5.6)	U = − 1.046	0.296
Number of painful days the past week - postoperatively	1.0 ± 2.1 (0.3–1.7)	1.5 ± 2.7 (0.6–2.3)	U = − 0.826	0.409
Mean reduction painful days	∆ = 3.5	∆ = 3.4	U = − 0.095	0.924
Test statistics	W = – 5.290	W = – 5.261		
p value	*p* < 0.001	*p* < 0.001		
FFI-D points preoperatively	88.4 ± 25.0 (74.9–91.9)	86.1 ± 27.7 (74.1–95.1)	U = − 0.547	0.584
FFI-D points postoperatively	28.9 ± 12.5 (24.7–33.1)	37.9 ± 26.4 (29.4–46.4)	U = − 0.740	0.460
Mean reduction FFI-D points	∆ = 59.5	∆ = 48.2	U = − 0.834	0.404
Test statistics	W = − 5.874	W = − 5.918		
p value	*p* < 0.001	*p* < 0.001		

Significance at *p* < 0.05

*W* Wilcoxon test, *U* Mann–Whitney U test, *SD* Standard deviation, *CI* Confidence interval, *∆* mean difference

**Table 5:** Clinical outcome

	Minimally invasive surgery (MIS)	Open surgery (OS)	Test statistics	*p* value
Number of patients	40	44		
Clinical axis correction (complete/satisfactory/inadequate)	30/8/2	30[1]/11[1]/2	χ² = 0.366	0.833
Persistent shoe conflict	3	4	χ² = 0.132	0.716

Significance at *p* < 0.05

*χ²* Chi-square

[ ]: one patient with different results in two corrected lesser toes

**Table 6:** Radiological follow-up of the surgical techniques in lesser toe surgery 18 months postoperatively

	Minimally invasive surgery (MIS)	Open surgery (OS)	Test statistics	*p* value
Insufficient axis correction	2	2	χ² = 3.862	0.145
Good dp alignment	35	36		
Tolerable dp alignment	3	6		
Inadequate dp alignment	2	2		
Good dp alignment	29	33		
Lateral deviation	8	8		
Medial and lateral deviation (multiple rays)	0	1		
Medial deviation	3	2		
Inadequate ml alignment	2	2		
Complete osseous consolidation	40	36	χ² = 7.111	**0.029**
Partial osseous consolidation	0	2	χ² = 7.111	**0.029**
Non union	0	6	χ² = 6.165	**0.013**

Significance at *p* < 0.05

*χ²* Chi square, *dp* dorsoplantar, *ml* mediolateral

**Table 7:** Safety of the surgical techniques in lesser toe surgery

Complications	Minimally invasive surgery (MIS)	Open surgery (OS)	Test statistics	*p* value
Intraoperatively	0	0	*	*
Incompliance	7	8	χ² = 0.056	0,813
Type of wire complications	4	3	χ² = 7.036	**0.030**
Wire buckling	0	3	χ² = 3.033	0.082
Wire migration	4	0	χ² = 4.265	**0.039**
Toe ischemia	0	1	χ² = 0.990	0,320
Toe necrosis	0	0	*	*
Toe loss	0	0	*	*
Impaired wound healing	1	7	χ² = 4.777	**0**,**029**
Superficial infection	0	5	χ² = 5.168	**0.023**
Osteitis	0	0	*	*
Neuropathic pain syndrome	3	3	χ² = 0.001	0.979
Arthrofibrosis MTP-joint	2	2	χ² = 0.000	0.983
Keloid or stricture	0	2	χ² = 1.906	0,167
Thromboembolism	0	0	*	*

Significance at *p* < 0.05

*U* Mann–Whitney U, * incalculable

## Discussion

To the best of our knowledge, this is the first randomised clinical trial with an adequate sample size and a mean follow-up of 18 months to compare the minimally invasive and open surgical approaches for lesser toe corrections. The study design corresponds to a real-world study, including further co-pathologies of the first ray or midfoot, which were treated in parallel without significantly affecting the comparability of the two groups. Our main results demonstrate comparable subjective, functional, and clinical patient outcomes for lesser toe corrections using the open surgical (OS) and minimally invasive surgical (MIS) techniques, while proving a significantly lower risk of soft tissue and bone healing complications in the MIS group.

Surgical treatment options for lesser toe deformities, including MIS techniques, are considerably less frequently addressed in the literature than surgery for the first ray or metatarsalgia [10]. This is particularly true when focusing on comparative studies of MIS versus open techniques [3–5].

The comparative study by Yassin et al. (2017) included 352 patients with hammertoe deformities [5]. OS was performed as proximal interphalangeal joint resection arthroplasty with K-wire fixation (265 patients with 454 toes). MIS was performed using tendon release and percutaneous diaphyseal osteotomy of the middle and proximal phalanx, followed by a three-week period of tape dressing. Thus, OS using resection arthroplasty was not comparable to the arthrodesis approach in the present study. The only difference between MIS and the current study was the postoperative fixation regime, which did not involve K-wire transfixation, instead using Coban taping. The retrospective comparative study by Mateen et al. (2021) included 41 patients [4]. The MIS group consisted of 54 feet and 124 toes. Their surgical technique included soft tissue release and percutaneous denudement of all cartilage in the proximal interphalangeal joint using a burr, followed by retrograde insertion of a 2.5 mm cannulated screw for arthrodesis fixation. OS was performed on 14 feet (22 toes), involving extensor tendon lengthening, head resection of the proximal phalanx, and release of the MTP joint, with K-wire fixation. Except for the arthrodesis concept, this method used similar surgical steps to the MIS technique, as described in the present study. Further studies have compared the Weil osteotomy with the DMMO procedure [6], or with a combined procedure involving the MIS correction of the lesser toes and percutaneous metatarsal diaphyseal osteotomies [3].

Thus, the manifold surgical technical variants and combinations of methods, which depend on the severity and flexibility of the lesser toe deformity, certainly limit the direct comparability of these studies with the current study, which focused strictly on surgical correction at the lesser toe level.

However, what is common to most studies of surgical lesser toe correction is the favourable overall outcome. Yassin et al. reported similar patient satisfaction rates for OS and MIS in their 2017 comparative study on hammertoe correction, but did not quantify this statement [5]. In open surgery, regardless of the specific surgical technique or mode of fixation, satisfaction rates of between 84% and 92% have been reported [11–15]. Similarly, the current study found that overall patient satisfaction was high, at over 92%. Furthermore, 89% of patients said they would undergo the procedure again if necessary. Regardless of the surgical technique, more than 90% would recommend the procedure to other patients, or even family members. These results are comparable with those of Carvalho et al., who focused on percutaneous second toe deformity correction via MIS and found that 88.4% of patients were satisfied, with 95% saying they would undergo surgery again [16].

Patient-related outcome measurements (PROMs) are a common method of evaluating postoperative results [17–19]. Due to the reliability and validity of our patients’ native languages, and the mean time needed to establish a score of eight minutes [11], we decided to use the FFI score [8]. Of the four comparative studies on lesser toe surgery or metatarsalgia that we are aware of, Yeo et al. used AOFAS and RAND 36 scores to evaluate their functional results [6]. They observed significant improvements over time, but found no difference between the two surgical techniques (postoperative *p* = 0.831). This aligns well with the findings of the current study. Carvalho et al. reported a doubling of AOFAS values, from 47.5 to 95.7, following MIS of the second toe [12]. Other studies investigating clinical results after OS of the lesser toes found good to excellent results during follow-up [13–16]. This could also be confirmed for MIS [17–21]. In the current study, we observed significant improvements in the FFI-D of 59.5 (MIS) and 58.4 (OS), respectively. This corresponds to an improvement of more than three times the minimally clinically relevant difference (MCID) [11]. As in the aforementioned studies, both surgical techniques for lesser toe correction appear to deliver the desired functional results, even though the PROMs used were not fully comparable.

With respect to the clinical assessment of toe alignment in the current study, 80 out of 84 patients (95.2%) had good to excellent axis correction. Two patients in the MIS group and two in the OS group showed recurrent deformities during follow-up (*p* = 0.883), despite there being more claw toe deformities in the OS group preoperatively. Persistent shoe conflicts (Table 5) and scar strictures or cases of arthrofibrosis (Table 7) were very rare, with no difference observed between the two cohorts (*p* >0.05), indicating that both techniques were equally capable of achieving an adequate axis correction. We used K-wire fixation exclusively to maintain the correction in both groups. Yassin et al. found recurrent deformities in 6.2% of cases, with an indication for revision surgery in 2.6% of cases [5]. Mateen et al. documented four cases of recurrent deviation in the MIS group, with no such cases in the OS group. Due to the small sample size, this difference was not statistically significant [4]. Yeo et al. found much better postoperative MTP joint mobility after MIS than after Weil osteotomy (*p* = 0.043), most likely due to the extra-articular location of the osteotomy [6]. Kramer et al. found recurrent deformities in 5.6% of their 2,698 open lesser toe surgeries. In 3.5% of cases, this led to revision surgery [22]. Other studies have reported rates of up to 17% for persistent lesser toe deformities following open surgery. MIS has shown good clinical results, with recurrence rates ranging from 0 to 3.7% [17, 21, 23, 24]. Stiffness does obviously occur following MIS as well [12]. Thus, both surgical techniques appear to produce good clinical results in lesser toe correction.

Radiological control after 18 months showed that more than 92% of corrected toes exhibited successful osseous healing and anatomical alignment. There were two manifest recurrent deformities in each cohort (*p* = 0.593) during the follow-up period. Therefore, the total recurrence rate was 4.8%, which is consistent with the aforementioned studies. A statistically significant difference was found between the two study groups in the bone healing process. Six of the 44 OS patients developed a non-union radiographically, while none of the MIS patients experienced any bone healing complications. This may be due to the location and type of osteotomy. In the OS cohort, the osteotomy is articular-sided with complete separation, whereas in the MIS group, one cortex generally remains intact at the junction of the diaphysis and metaphysis. The vascular supply may also play a vital role in this context, and it is assumed that this is better preserved in the MIS technique.

Mateen et al. found comparable bone healing periods in their MIS and OS groups (*p* = 0.065) [4]. Yeo et al. documented a 100% consolidation rate for all their patients, which aligns with the findings of Ray et al. [25]. Other studies regarding OS in lesser toe surgery found fusion rates ranging from 81 to 100% [13, 15, 26, 27]. MIS generally led to full bony consolidation in the lesser toes, with non-unions occurring only in isolated cases [5, 17, 19, 21, 23]. These data suggest that MIS can provide axis correction comparable with OS while presenting a lower risk of complications in bone healing.

Fifteen of the 95 patients were labelled as non-compliant postoperatively, which usually meant that they did not follow the partial weight-bearing regimen within the therapy shoe. This resulted in four wire migrations in the MIS group and three wire deformations in the OS group, representing a significant difference between the two groups (*p* = 0.03). This may be due to the different positioning of the wire in the two groups. With OS, the wire was within the distal metatarsal bone; with MIS, wires were placed in the basal phalanx only. Overall, wire complications were observed in 7.4% of cases. Mateen et al. reported 4% of unexpected screw removals in their minimally invasive cohort and no wire issues in their open surgical cohort [4]. Yassin et al. reported a 5.5% incidence of wire migration in their open surgical group. In the MIS group, taping was used for fixation [5]. McKenzie et al. conducted a large retrospective study of 2,017 open surgical treatments for lesser toe deformities. They found a 1.14% rate of non-infection-based wire migration in all cases [28]. In summary, implant migration appears to be a rare phenomenon, with specific risks associated with each implant.

Another notable complication is ischaemia. We had one case of postoperative transient ischaemia in the OS group, which was resolved by reducing the toe length on the wire. No toes were lost due to postoperative ischaemic problems. Yassin et al. reported 0.5% ischaemic complications resulting in toe amputation in 0.25% of cases [5]. Of the 1,000 hammer toes corrected in OS in the retrospective study by Kramer et al., 0.6% suffered from ischaemia, and 0.4% of cases ended in toe amputation [22]. These complications are rare, consistent with the current findings.

Soft tissue healing is a parameter of paramount importance for the success of lesser toe surgery, as complications in this area may lead to severe consequences such as surgical site infections. In our study, seven patients with OS (7.4%) developed impaired and prolonged soft tissue healing, resulting in five superficial wound infections (5.3%). The MIS group contained one such case, with no subsequent deep infection. Consistent with our data, Yassin et al. found 7% wound complications and 4% wound infections in the OS group. Surprisingly, they also found a high 20% of cases with compromised soft tissue healing and 2.3% surgical site infections in the MIS group [5]. Mateen et al. reported a 0.8% wound complication rate in the MIS group and a 4.6% rate in the OS group, with no deep infections [4]. Other non-comparative studies have shown infection rates ranging from 0.3% to 4.9% for OS of lesser toe deformities [22, 28], and rates of infection ranging from 0% to 13% for MIS [23, 24, 29]. Maidmann et al. described how comorbidities such as diabetes mellitus, chronic obstructive pulmonary disease, and osteoporosis could increase the risk of wound infection following lesser toe surgery by up to fourfold [30]. In conclusion, MIS appears to be less susceptible to impaired soft tissue healing (*p* = 0.029) and wound infection (*p* = 0.023), despite the potential for higher local temperatures during the use of a high-speed burr during surgery, and the associated risk of heat necrosis, which has been reported in the early stages of the learning curve [1, 2].

Further types of complications have been reported sporadically. Richmann et al. observed isolated cases of long-lasting local hypesthesia, peroneal nerve neuritis, and persistent local pain syndrome in their study of 99 patients with OS [31]. Other studies investigating MIS results for hammertoe deformities did not identify any neuropathic complications [23, 24], whereas we found three patients in each cohort presenting with neuropathic pain after an 18-month follow-up period (*p* = 0.2).

The main limitations of the current study are its monocentric design and possible bias due to confounders, as lesser toe deformities were treated alongside co-pathologies of the foot in most cases (79%). Further limitations include the non-blinded clinical evaluation, in contrast to the blinded radiological evaluation, and the relatively short mean follow-up period of 18 months. Even though it is one of the most frequently used assessment instruments in foot and ankle surgery, the FFI scoring system has known weaknesses, such as the lack of documentation of joint mobility and stability, and the evaluation of toe positioning and footwear [32]. In future, multicentre studies with larger patient cohorts and longer follow-up periods should be conducted to confirm our findings. Furthermore, other validated scoring systems such as the EFAS or FAOS (Foot and Ankle Outcome Score) score could be employed to achieve greater comparability and reach a broader audience [11]. For evaluating radiological outcomes, computed tomography would be the most precise tool, even though this is not standard practice in lesser toe surgery.

## Conclusion

Both MIS and OS techniques for correcting lesser toe deformities were found to be safe and reliable procedures, achieving high levels of patient satisfaction. Clinical, radiological, and functional outcomes were comparable for both study groups. MIS appeared to have a significantly better risk profile with regard to soft tissue and bone healing than OS. While this may favour the MIS technique, OS for lesser toe deformities is widespread and MIS is not available everywhere. Ultimately, the decision lies with the experienced surgeon, who should make an individual decision for each patient based on their personal expertise.

## Data Availability

No datasets were generated or analysed during the current study.
